# High-Concentration Insulin Glargine Overdose: Polyphasic Patterns of Blood Insulin Levels

**DOI:** 10.7759/cureus.52410

**Published:** 2024-01-16

**Authors:** Hitomi Tsunashima, Hiroaki Takada, Hiroki Shiojima, Hisashi Yoneyama, Eijyu Hasegawa

**Affiliations:** 1 Emergency Medicine, National Disaster Medical Center, Tokyo, JPN; 2 Emergency Medicine, National Defense Medical College, Saitama, JPN; 3 Critical Care Medicine and Trauma, National Disaster Medical Center, Tachikawa, JPN

**Keywords:** u300 glargine, insulin overdose, lantus, glargine, high concentration insulin

## Abstract

In the treatment of diabetes mellitus, there is a growing trend towards using high-concentration insulin, with Lantus XR (Bridgewater, NJ: Sanofi-Aventis U.S. LLC), which has a drug concentration three times higher than that of conventional Lantus (100 U/mL; Bridgewater, NJ: Sanofi-Aventis U.S. LLC), being a prominent example. This type of high-concentration insulin is known for its smaller injection volumes, leading to a slower absorption rate and maintenance of more consistent blood insulin levels. When administered in high doses, the pharmacological effects of insulin are generally prolonged; however, insulin glargine overdose rarely occurs, and its pharmacokinetics remain unclear. We encountered a case of an insulin overdose in a 19-year-old female patient, who had self-injected glargine (Lantus XR) 1,350 units and aspart (NovoRapid; Bagsværd, Denmark: Novo Nordisk A/S) 600 units. We measured blood glucose and insulin levels over time. Bimodal peaks in blood insulin levels were observed, and we adjusted high doses of intravenous infusion with a 50% glucose solution until the blood insulin levels returned to the normal range. Consequently, the patient was treated without inducing severe hypoglycemia. U300 glargine overdose may lead to both a multimodal elevation in blood insulin levels and prolonged hypoglycemia compared to U100 glargine. Therefore, monitoring blood insulin levels and adjusting treatment accordingly may contribute to safer patient management. This study represents the initial documentation of blood insulin levels measured in a U300 glargine overdose patient, revealing a bimodal peak.

## Introduction

High-concentration insulin formulations are increasingly used in the management of diabetes mellitus [1]. Lantus XR (Bridgewater, NJ: Sanofi-Aventis U.S. LLC) is a 300 U/mL insulin glargine preparation (U300 glargine) with a drug concentration three times higher than that of conventional Lantus (100 U/mL). Use of high-concentration insulin allows the injection of drugs in smaller volumes and reduces the surface area, resulting in slower absorption and potentially flatter and more sustained blood insulin. Although the pharmacological effects of insulin are believed to be prolonged when administered in large doses, insulin glargine overdose rarely occurs, and its pharmacokinetics remain unclear [2]. We measured the blood insulin levels in a case of U300 glargine overdose for the first time, providing possible insights for assessing the treatment.

## Case presentation

A 19-year-old female with a history of type 1 diabetes was found unconscious and transported to our hospital. Empty containers of U300 glargine (Lantus XR) 1,350 units and aspart (NovoRapid; Bagsværd, Denmark: Novo Nordisk A/S) 600 units were found; hence, the patient was presumed to have self-injected these drugs to several sites of her abdomen. Upon contact with the emergency medical service (EMS) personnel, the patient had a Glasgow Coma Scale (GCS) score of 3 but showed stable respiratory and circulatory status. Her blood glucose level was below the measurement sensitivity, and 40 mL of a 50% glucose solution was intravenously administered by the EMS personnel. Upon arrival at the hospital, the patient’s GCS score remained at 3, and the blood glucose level was low (45 mg/dL); thus, another 40 mL of 50% glucose solution was intravenously administered. The blood glucose level subsequently increased (150 mg/dL), and the GCS score increased to 6 (E1V1M4). Physical examination revealed no notable findings. Initial blood tests showed an increase in inflammatory response (white blood cell count: 16,400/µL and C-reactive protein: 0.1 mg/dL), severe hypoglycemia (39 mg/dL), and a high hemoglobin A1c level (10.2%). No electrolyte abnormalities or hepatic or renal dysfunctions were observed. The patient was eventually diagnosed with a hypoglycemic coma due to an insulin overdose and was admitted to the intensive care unit (ICU).

After admission to the ICU, hypoglycemia (blood glucose level <70 mg/dL) frequently occurred and repeated intravenous bolus administration of glucose solution was required. Therefore, the patient’s treatment was switched to continuous 50% glucose solution infusion via the central venous route. Blood glucose was measured every hour, and the infusion rate was adjusted accordingly. Gradual improvement in speech and body movement was observed 12 hours after admission, and the patient regained consciousness on day two. Oral intake (1,600 kcal/day) was initiated on day three.

To assess the metabolic status of the injected insulin, blood insulin was also monitored. The blood insulin level was 621.0 µU/mL at the time of admission and seemed to decrease over time; however, it showed a bimodal peak of 1845.8 µU/mL and 2648.5 µU/mL on days one and two, respectively. We initially planned to reduce the infusion rate of 50% glucose solution in response to the rising trend in blood glucose levels. However, confirming the re-elevation of blood insulin levels, glucose infusion was not immediately reduced, and instead, a relatively high dose was administered until insulin levels were normalized.

**Figure 1:**
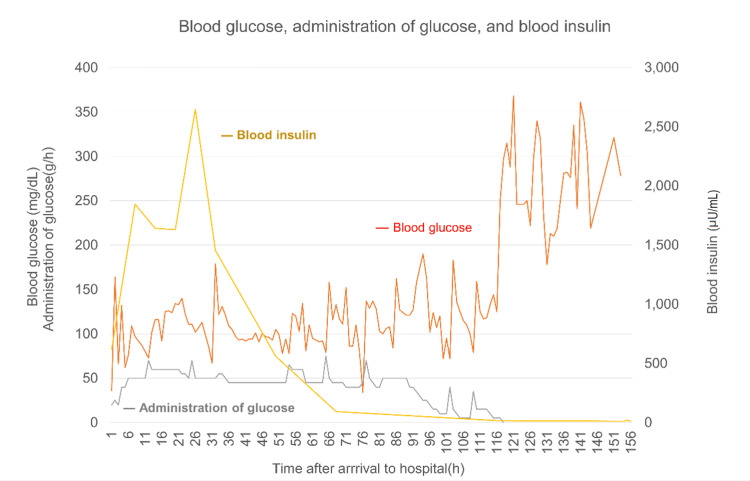
Changes in blood glucose levels, administration of glucose, and blood insulin concentrations. Blood glucose was measured every hour, and the infusion rate of the 50% glucose solution was adjusted accordingly. The blood insulin level was 621.0 µU/mL at the time of admission and increased to 2648.5 µU/mL on day two. As the blood insulin levels tended to decrease, the infusion rate of 50% glucose solution was decreased gradually. The blood insulin level returned to normal on day six and the 50% glucose infusion was discontinued.

By day six (117 h after admission), the blood insulin level returned to normal, and the 50% glucose infusion was gradually decreased and discontinued. Changes in blood glucose levels, administration of glucose, and blood insulin concentrations are illustrated in Figure 1. Hypoglycemia did not recur, and subcutaneous injection of normal-dose insulin was resumed on day seven. After psychiatric clearance, the patient was discharged on day 11 without any complications.

## Discussion

This study suggests that in cases of U300 glargine overdose, polyphasic peaks in the blood insulin levels may be observed, hence monitoring blood insulin levels and adjusting treatment appropriately may safely guide hypoglycemia correction. This study is significant, as it provides the initial documentation of blood insulin levels measured in a U300 glargine overdose patient, revealing a distinct bimodal peak.

The duration of the effect of insulin glargine is reported to be approximately 24 hours when administered in therapeutical doses (0.3-0.5 units/kg). However detailed information about the duration and strength of effect when overdosed, such as in the present case (27 units/kg), remains unknown.

Several studies, including a systematic review, have reported on insulin overdoses, suggesting that they can cause prolonged, refractory hypoglycemia [3]. This is particularly evident with long-acting insulin, such as glargine, owing to its delayed absorption from the injection site and possibly prolonged clearance due to the depot effect.

A total of 23 cases of subcutaneous insulin glargine overdose were found in PubMed (searched by the keywords "Lantus, glargine, overdose"), as detailed in Table 1, with only one involving U300 glargine (no. 23 in Table 1). We initially hypothesized that U300 glargine overdose may result in prolonged hypoglycemia compared with U100 glargine overdose, owing to its higher concentration and slower absorption. However, in our case and case no. 23 in Table 1, no remarkable difference was observed compared with U100 glargine cases. Overall, as the insulin dosage increased, the treatment duration seemed to become longer, but no clear pattern could be identified. We could not find any other correlation or pattern between the cases.

**Table 1:** Cases of subcutaneous insulin glargine overdose. Numbers 1-23 are the cases of subcutaneous insulin glargine overdose found in the literature. Only one of the 23 cases included U300 glargine (no. 23). No remarkable difference was observed in case No. 23 and this case (X), compared with U100 glargine cases (no. 1-22). Treatments other than glucose infusion were administration of glucagon, octreotide, and steroids, subcutaneous depot removal, and oral consumption of high-carbohydrate meals. F: female; M: male; U: unknown; A: aspart, L: lispro; N: novorin; G: glucagon; O: octreotide; S: steroid; OI: oral intake of high-carbohydrate foods or drinks; DE: depot excitation; X: case in this article; DM: diabetes mellitus

No.	Age	Sex	DM	Type	Glargine (U)	Other types of insulin(U)	IV (h)	Other treatments	Author	Published year
1	33	F	N	-	300	A 200	40	OI	Tofade and Liles [4]	2004
2	21	F	Y	U	26	-	60	-	Brvar et al. [5]	2005
3	41	M	Y	2	180	-	144	-	Tsujimoto et al. [6]	2006
4	22	F	Y	1	300	A 300	59	OI	Fromont et al. [7]	2007
5	31	F	N	-	1,000	-	130	OI	Ashawesh et al. [8]	2009
6	37	M	Y	U	150	-	48	-	Fuller et al. [9]	2009
7	29	F	Y	U	640	-	72	OI	Ohama et al. [10]	2009
8	76	M	Y	U	500	-	96	-	Doğan et al. [11]	2012
9	51	F	Y	U	2,700	-	120	OI	Lu et al. [12]	2011
10	39	M	Y	2	3,800	L 800	81	-	Mork et al. [13]	2011
11	12	F	N	-	2,000	-	130	OI	Kumar et al. [14]	2012
12	26	M	Y	1	4,800	-	120	DE	Warriner et al. [15]	2012
13	56	M	Y	2	3,300	-	115	O	Groth et al. [16]	2013
14	38	F	Y	U	300	N 200	58	OI	Uesugi et al. [17]	2014
15	35	M	Y	U	300	-	48	OI	Tsujino et al. [18]	2014
16	45	M	Y	2	3,600	-	120	G	Karatas et al. [19]	2015
17	46	F	Y	2	900	L 2,100	141	OI	Ishibashi et al. [20]	2015
18	43	M	Y	1	1,500	A 600	115	-	Kim et al. [21]	2016
19	43	M	Y	1	900	A 900	96	-	Kim et al. [21]	2016
20	25	F	Y	1	1,200	-	96	-	Nakamura et al. [22]	2017
21	36	F	Y	2	10,000	-	150	G, O, S	Tariq et al. [23]	2018
22	27	F	Y	2	900	-	96	-	Sandooja et al. [24]	2020
23	45	F	Y	1	4,050	L 300	144	G	Endall et al. [1]	2020
X	19	F	Y	1	1,350	A 600	119	-	-	2022

This discrepancy may be attributed to various factors that affect insulin pharmacokinetics, including the site of administration, whether it was injected in a single or multiple sites, injection volume, solution concentration, and certain conditions of injection sites, such as lipodystrophy or amyloidosis. Additionally, the variety of treatment methods employed, including the administration of glucagon, octreotide, and steroids, subcutaneous depot removal, and oral consumption of high-carbohydrate meals, may also have affected the treatment duration. Therefore, it can be difficult to predict the persistence of hypoglycemia in cases of insulin overdose just by the type and amount of insulin administered.

Several studies propose that monitoring fluctuation in blood insulin levels is crucial for determining the duration of possible hypoglycemia and optimal treatment [13,21]. While these studies have measured blood insulin levels in cases of U100 glargine overdose, none have reported on U300 glargine, making this the first case. In our patient, bimodal peaks in insulin levels were observed at 7 and 25 h after injection. Therefore, instead of immediately decreasing the glucose infusion rate based on blood glucose levels, we confirmed the normalization in insulin levels in advance. This bimodal behavior of blood insulin was similar to the report of a patient who experienced insulin glargine (U100) and aspart overdose [21]. The mechanism of this behavior is unknown, although it is thought to be affected by the variation in injection sites, conversion speed from glargine to its metabolically active form, and site-specific circulation. U300 insulin may have made it more prone to inducing this biphasic pattern, due to the slower absorption rate. However, there is limited data on blood insulin levels in both U100 and U300 insulin, making comparison difficult.

## Conclusions

Overdosing on high-concentration insulin, such as U300 glargine, may lead to sustained or multiple peaks of elevated insulin in the bloodstream, resulting in an extended period of hypoglycemia than initially expected. Frequent measurement of blood insulin levels may help recognize fluctuations and guide the appropriate rate and duration of glucose infusion. In the present case, although blood glucose level was within normal levels, we observed the re-elevation of blood insulin and extended the glucose infusion therapy, successfully treating the patient without inducing severe hypoglycemia. Consequently, monitoring blood insulin levels may be crucial to enhance safety in patient treatment. Further investigations into similar cases are essential to advance the treatment of U300 glargine insulin overdoses, leading to safer and more effective patient care.
